# Nonislet Cell Tumor Hypoglycemia in a Patient with Adrenal Cortical Carcinoma

**DOI:** 10.1155/2016/5731417

**Published:** 2016-11-10

**Authors:** Se Won Kim, Seung-Eun Lee, Young Lyun Oh, Seokhwi Kim, Sun Hee Park, Jae Hyeon Kim

**Affiliations:** ^1^Division of Endocrinology, Department of Internal Medicine, Sahmyook Medical Center, Seoul, Republic of Korea; ^2^Department of Medicine, Samsung Medical Center, Sungkyunkwan University School of Medicine, Seoul, Republic of Korea; ^3^Department of Pathology, Samsung Medical Center, Sungkyunkwan University School of Medicine, Seoul, Republic of Korea; ^4^Graduate School of Medical Science and Engineering, Korea Advanced Institute of Science and Technology, Daejeon, Republic of Korea

## Abstract

Nonislet cell tumor hypoglycemia (NICTH) is a rare but serious paraneoplastic syndrome in which a tumor secretes incompletely processed precursors of insulin-like growth factor-II (IGF-II), causing hypoglycemia. Here, we report an exceptional case of NICTH caused by nonfunctioning adrenocortical carcinoma in a 39-year-old male with recurrent hypoglycemia. The patient's serum IGF-II/IGF-I ratio had increased to 27.8. The serum level of the IGF-II/IGF-I ratio was normalized after removal of the tumor, and the hypoglycemic attacks no longer occurred after the operation.

## 1. Introduction

Hypoglycemia is a common medical emergency that can sometimes be the first manifestation of tumor disease [1]. Hypoglycemia can be caused by several tumors, including islet and nonislet tumors. Nonislet cell tumor hypoglycemia (NICTH) is a rare but serious complication of malignancy [2, 3]. It has been estimated that NICTH is four times less common than insulinoma [4]. It is believed that insulin-like growth factor-II (IGF-II, 7.5 kDa) or its high-weight precursor (“Big”-IGF-II, 10–20 kDa) produced by tumors is the primary hormonal mediator of NICTH. In 1988, Daughaday et al. reported the first description of NICTH due to aberrant expression of an immature form of IGF-II in a patient with leiomyosarcoma [5]. In a previous extensive review of this topic in 2007, De Groot et al. reported that, of the IGF-II-producing tumors causing hypoglycemia, 41% had a mesenchymal origin, 43% had an epithelial origin, 1% had a neuroendocrine and hematopoietic origin, and 14% had an unknown origin [6]. The tumors are typically large and slow growing.

NICTH has been rarely reported in cases of adrenocortical carcinoma. In this report, we describe a case of adrenocortical carcinoma with recurrent hypoglycemia that was diagnosed as NICTH associated with the production of IGF-II.

## 2. Case Presentation

A 39-year-old male patient initially presented to his general practitioner in May 2015 with nonsensical ramblings, sweating, and light-headedness. His past medical history consisted of hypertension a year earlier, but there was no other relevant history. His plasma glucose level was 40 mg/dL. The patient was referred to our hospital for further evaluation. His height was 177.1 cm and body weight was 64.0 kg. On physical examination, the patient appeared to be well developed and well nourished, and there were no signs of an excessive production of adrenocortical steroids. He had blood pressure of 130/86 mmHg, pulse of 73/min, and respiratory rate of 18/min. When the patient developed hypoglycemia, his serum insulin concentration was 1.7 *μ*IU/mL, C-peptide was 0.04 ng/mL, and blood glucose was 40 mg/dL. The patient's insulin antibodies were not detected. We confirmed significant hypoglycemia with suppression of endogenous insulin secretion.

**Figure 1:**
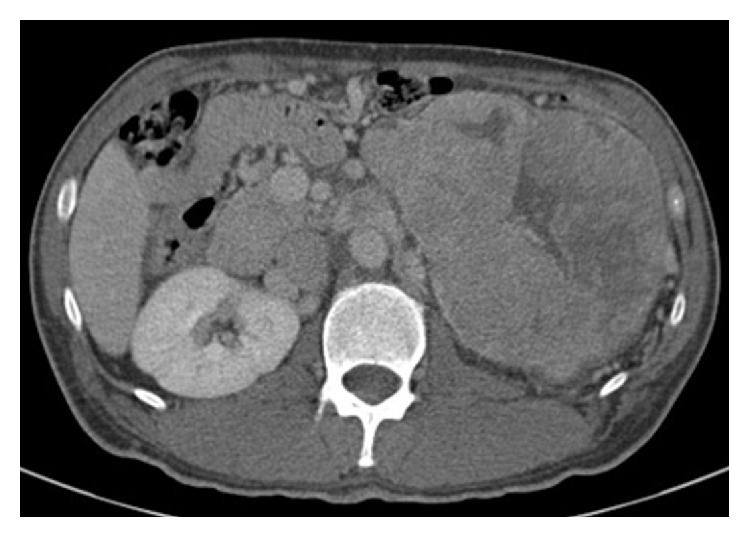
Abdominal computed tomography demonstrating a heterogeneously enhanced mass, 14 × 12 cm in diameter, with internal necrotic changes in the left adrenal region.

A 14-centimeter left adrenal mass was found on abdominal computed tomography (CT), obtained as part of the evaluation for recurrent hypoglycemia (Figure 1). The mass was solid, but partly cystic, and the findings of these examinations suggested that the mass may have had a necrotic lesion in the center. CT of the chest revealed no evidence of metastasis. On further questioning, he did not experience traditional attacks of pheochromocytoma, such as headaches, flushing, or palpitations. Urea and electrolytes, aldosterone, plasma renin activity, and testosterone were normal. A complete blood count with platelet count and measurement of serum calcium, creatinine, alanine aminotransferase, and aspartate aminotransferase levels were normal. Glycosylated hemoglobin (HbA1c) was 4.8%. The patient's 24-hour urine vanillylmandelic acid (VMA), metanephrine, and normetanephrine levels were normal. However, urinary epinephrine, norepinephrine, and cortisol levels were elevated. These results likely indicate compensation for hypoglycemia. The serum level of IGF-II was within normal range (555 ng/mL; normal, 288–736 ng/mL), but serum IGF-I level was decreased to 20 ng/mL (normal, 49–642 ng/mL). The serum IGF-II/IGF-I ratio had increased to 27.8 (normal, <10), suggesting a diagnosis of NICTH. The laboratory data on admission are shown in Tables 1 and 2.

**Figure 2:**
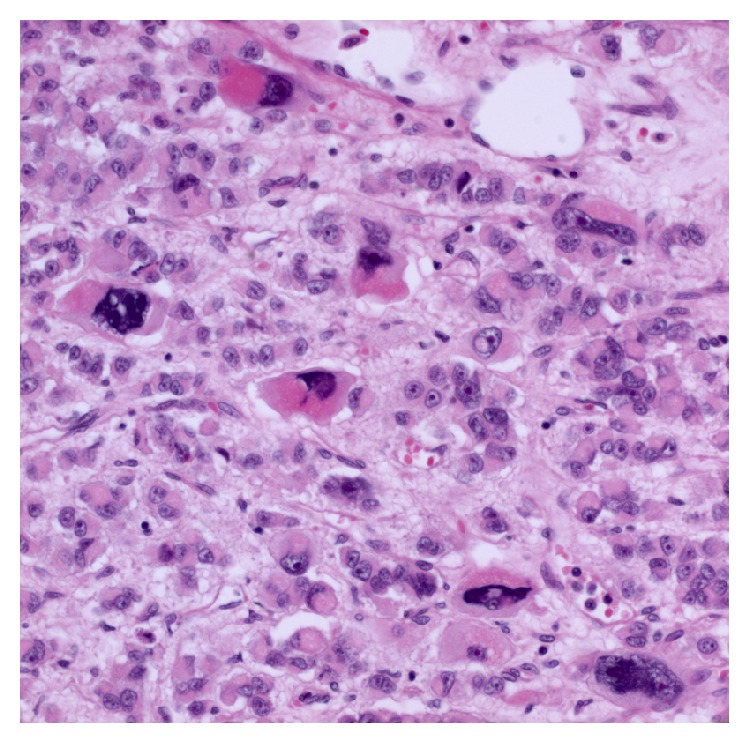
Histopathology showing malignant cells in the adrenocortical site (HE stain, ×400), with abundant eosinophilic cytoplasm, enlarged hyperchromatic nuclei, and prominent nucleoli. Several anaplastic cells were also shown in the field.

During hospitalization, symptoms associated with hypoglycemia occurred frequently, mostly in the morning, and required continuous hypertonic glucose infusion (220 g per day by intravenous administration). A left adrenalectomy, left nephrectomy, and left renal vein thrombectomy were performed, with the histopathology confirming an adrenocortical carcinoma measuring 18 × 16 × 9 cm. Macroscopically, the mass was closely attached to renal capsule but direct invasion into renal parenchyme was not identified. The cut section showed round and lobulated soft pinkish mass with multifocal hemorrhage and necrosis. Microscopic examination of the adrenal tumor revealed high nuclear grade (Fuhrman grade IV), 13 mitoses per HPF, vascular invasion, and necrosis. The clear cells accounted for less than 25% in the tumor (Figure 2). Based on the above findings, the pathological diagnosis of the adrenocortical carcinoma was made according to the diagnostic criteria of malignancy of adrenocortical tumors defined by Lau and Weiss [7]. Immunohistochemistry showed the tumor cells to be positive for inhibin-*α*, calretinin, and negative for chromogranin A.

He received adjuvant treatment with mitotane and replacement therapy with hydrocortisone postoperatively. After tumor excision, serum levels of glucose, insulin, and C-peptide normalized. The patient's postoperative IGF-I and IGF-II/IGF-I ratio levels returned to the normal range (188 ng/mL and 3.2, resp.), and he experienced no further hypoglycemic episodes. The follow-up abdominal CT was done 6 months after the operation, which revealed multiple hepatic metastases. We considered additional combined chemotherapy, but he was lost to follow-up.

## 3. Discussion

NICTH is a rare paraneoplastic syndrome and is the second most common cause of tumor-related hypoglycemia following insulinoma. In the review by Bodnar et al. [3], there were nearly 290 cases of NICTH reported in the English language medical literature in the past 25 years. This condition is usually associated with slow-growing and mesenchymal tumors such as sarcomas, fibromas, and mesotheliomas. Among primary epithelial tumors, episodes of hypoglycemia were described in patients with hepatocellular carcinoma, gastric cancer, renal cell carcinoma, phyllodes tumor of breast, and ovarian adenocarcinoma [3].

Adrenocortical carcinoma (ACC) is a rare malignancy with an annual incidence of 1-2 per million population [8]. The natural clinical course of ACC is not well known because of its poor prognosis. Only four cases of NICTH accompanied by ACC were recognized in a PubMed search from 1984 onward [9–12]. When we consider that many ACCs are associated with IGF-II overexpression, ACC might be a potential candidate causing NICTH through incomplete processing of pro-IGF-II [8].

No single pathogenetic mechanism can explain all cases of NICTH. However, the major cause of NICTH could be as follows: increased glucose utilization by large tumors, inhibition of glycogenolysis and gluconeogenesis from the liver, and suppression of counterregulatory hormones for insulin or insulin-like factors secreted by the tumor. NICTH is mediated via IGF-II, which exhibits a high degree of structural homology to proinsulin. In NICTH, 70% of the patients have high molecular weight IGF-II, so-called “big” IGF-II (10–20 kDa), although the total IGF-II values are within normal range [13]. “Big”-IGF-II forms binary complexes with IGF-binding protein (IGFBP), instead of the normal ternary complex, and these small binary complexes have greater capillary permeability and therefore increase IGF bioavailability to the tissues [14].

In the clinical setting, diagnosing NICTH is difficult because serum IGF-II levels are not always elevated in these patients. Hizuka et al. suggested that the IGF-II/IGF-I ratio in serum is useful for detecting IGF-II-producing NICTH [13]. They reported that the IGF-II/IGF-I ratio in serum exceeded 20 in patients with NICTH. In these patients, IGF-I production could be suppressed by an insulin-like factor, big IGF-II. Their data indicated that serum big IGF-II and IGF-II/IGF-I ratio are useful for screening patients with IGF-II-producing NICTH. However, assays for big IGF-II are not commercially available. Thus, the IGF-II/IGF-I ratio is used as a surrogate marker for big IGF-II concentration. A ratio of IGF-II/IGF-I > 10 indicates NICTH [15].

In NICTH, the best treatment for hypoglycemia is surgical resection of the tumor. The metabolic alterations caused by NICTH are fully reversible after successful surgical removal of a big IGF-II-producing tumor. In our case, we only measured total IGF-II including normal and big IGF-II and did not distinguish big IGF-II. Unfortunately, we could not investigate IGF-II expression in cancer tissues. The serum IGF-II level was not elevated, but the IGF-II/IGF-I ratio was increased to 27.8. The serum IGF-II/IGF-I ratio before surgery decreased and normalized after the removal of the tumor, and hypoglycemic attacks no longer occurred after the operation.

In conclusion, we have reported a very rare case of adrenocortical carcinoma associated with NICTH. We have found five cases, including our own case, of adrenocortical carcinoma causing NICTH during 30 years. The possibility of NICTH is suggested by low serum insulin and C-peptide during a hypoglycemic episode, with low IGF-I level. However, the circulating level of total IGF-II may be increased, decreased, or normalized, with an IGF-II/IGF-I ratio of 10 or more. In subjects with recurrent hypoglycemia and no history of diabetes, NICTH should be included in the differential diagnosis.

## Figures and Tables

**Table 1:** Endocrinological data from a patient with hypoglycemia.

	Levels	Normal range	Units
Blood glucose	40	70–109	mg/dL
Hemoglobin A1c	4.8	4–6	%
Insulin	1.7	1.1–11.2	*μ*IU/mL
C-peptide	0.04	0.69–3.59	ng/mL
Anti-insulin antibody	3.8	0–7	%
GAD II antibody	0.27	0-1	U/mL
IGF-I	20	49–642	ng/mL
IGF-II	555	288–736	ng/mL
ACTH	63.9	0–60	pg/mL
Cortisol	10	1.8–26.0	*μ*g/dL
DHEAS	359.2	34.9–479.4	*μ*g/dL

ACTH, adrenocorticotropic hormone; DHEAS, dehydroepiandrosterone sulfate; IGF, insulin-like growth factor.

**Table 2:** 24-hour urinary catecholamines and plasma metanephrines before surgery.

	Levels	Normal range	Units
Plasma metanephrines			
Metanephrine	0.12	<0.35	nmol/L
Normetanephrine	0.34	<0.64	nmol/L
24-hour urinary catecholamines			
Metanephrine	95.7	<229.5	*μ*g/day
Normetanephrine	168.2	<502	*μ*g/day
Norepinephrine	184.8	15–80	*μ*g/day
Epinephrine	32.0	0–20	*μ*g/day
VMA	5.4	<6.8	mg/day
Cortisol	180	14–97	*μ*g/day

VMA, vanillylmandelic acid.
